# Effect of Donepezil, Tacrine, Galantamine and Rivastigmine on Acetylcholinesterase Inhibition in *Dugesia tigrina*

**DOI:** 10.3390/molecules21010053

**Published:** 2016-01-11

**Authors:** Cristiane Bezerra da Silva, Arnildo Pott, Selene Elifio-Esposito, Luciane Dalarmi, Kátia Fialho do Nascimento, Ligia Moura Burci, Maislian de Oliveira, Josiane de Fátima Gaspari Dias, Sandra Maria Warumby Zanin, Obdulio Gomes Miguel, Marilis Dallarmi Miguel

**Affiliations:** 1 Department of Pharmacy, Federal University of Paraná (UFPR), Av. Pref. Lothário Meissner 3400, Jardim Botânico, Curitiba 80210-170, Brazil; ludalarmi@gmail.com (L.D.); ligia.burci@gmail.com (L.M.B.); maislian@gmail.com (M.O.); jodias@pop.com.br (J.F.G.D.); sandramariazanin@gmail.com (S.M.W.Z.); obdulio@ufpr.br (O.G.M.); dallarmi@ufpr.br (M.D.M.); 2 Department of Biology, Federal University of Mato Grosso do Sul (UFMS), Av. Senador Filinto Müller, Campo Grande 79046-460, Brazil; arnildopott@gmail.com; 3 Post-Graduation in Health Sciences, Pontíficia Universidade Católica do Paraná, Imaculada Conceição, 1155, Prado Velho, Curitiba 80215-901, Brazil; seleneesposito@gmail.com; 4 Department of Celular Biology, Federal University of Paraná (UFPR), Centro Politécnico, Jardim das Américas, Curitiba 81530-900, Brazil; kafialho@hotmail.com

**Keywords:** Alzheimer’s disease, anticholinesterasic, convulsant activity, spontaneous locomotion, planarian

## Abstract

*Dugesia tigrina* is a non-parasitic platyhelminth, which has been recently utilized in pharmacological models, regarding the nervous system, as it presents a wide sensitivity to drugs. Our trials aimed to propose a model for an *in vivo* screening of substances with inhibitory activity of the enzyme acetylcholinesterase. Trials were performed with four drugs commercialized in Brazil: donepezil, tacrine, galantamine and rivastigmine, utilized in the control of Alzheimer’s disease, to inhibit the activity of acetylcholinesterase. We tested five concentrations of the drugs, with an exposure of 24 h, and the mortality and the inhibition of acetylcholinesterase planarian seizure-like activity (pSLA) and planarian locomotor velocity (pLMV) were measured. Galantamine showed high anticholinesterasic activity when compared to the other drugs, with a reduction of 0.05 μmol·min^−1^ and 63% of convulsant activity, presenting screw-like movement and hypokinesia, with pLMV of 65 crossed lines during 5 min. Our results showed for the first time the anticholinesterasic and convulsant effect, in addition to the decrease in locomotion induced by those drugs in a model of invertebrates. The experimental model proposed is simple and low cost and could be utilized in the screening of substances with anticholinesterasic action.

## 1. Introduction

Alzheimer’s disease is known as a degenerative and progressive disorder of the brain and is considered the most common form of dementia of the elderly, especially in developed countries. The disease is associated with a loss of the cholinergic system with a reduction of the levels of acetylcholine in areas of the brain that deal with learning, memory, behavior and emotional responses [1]. Thus, the most promising approach for symptomatic treatment of Alzheimer’s disease is to increase the synaptic levels of acetylcholine in the brain through inhibition of acetylcholinesterase (AChE). The inhibitors of AChE, such as galantamine, donepezil, rivastigmine and tacrine, are the main medicines for clinical management of the disease and are widely applied in Brazil [2].

Different pharmacological models, mainly those using rodents, were proposed to select substances that could act as AChE inhibitors [3,4,5,6,7]. However, the cost of the acquisition and maintenance of animals *in vivo*, the need for an adequate physical structure, care in handling and minimizing suffering justifies the demand of alternative methods of low cost that are more accessible and that have a higher number of individuals, as well as to achieve results in less time than conventional trials.

Species of planarians have been shown to be extremely precise and relevant models in pharmacological studies, once they exhibit cephalization, including the brain, which has many characteristics in common with the nervous system of more specialized species [8]. Among such characteristics, the general morphology and physiology of the neurons, the presence of dendritic spines and the utilization of various neurotransmitters found in mammals, including humans, are also present in planarians [9].

The species *Dugesia tigrina* (Platyhelminthes, class Turbellaria) is a free-living flatworm and has a central nervous system with bilateral symmetry [10]. The presence of a characteristic and moderately simple central nervous system was recently proven on the basis of electroencephalography [11] and allows the easiness of the manipulation and comprehension of drug effects on this species, once its genes and neurotransmitters were found to be the same as those found in vertebrates [12,13]. Therefore, planarians are a promising model experimental organism for the investigation of biochemical and functional interactions among the receptor systems of neurotransmitters and can display the pharmacological action of several drugs [14,15,16,17].

Therefore, the objective of our work was to verify if the drugs galantamine, donepezil, Rivastigmine and tacrine inhibit the action of acetylcholinesterase in *D. tigrina*, aiming to establish an *in vivo* model for screening substances that could be applied in the control of Alzheimer’s disease.

## 2. Results and Discussion

Our results demonstrated that *D. tigrina* is sensitive to the tested drugs. Galantamine had more accentuated effects than the other drugs, with 100% mortality under a concentration of 25 μg·mL^−1^ and LC_50_ of 8.3 μg·mL. Considerable LC_50_ was also observed for rivastigmine (17.4 μg·mL^−1^) (Table 1).

**Table 1:** Mortality (%) and LC_50_ of *D. tigrina* submitted to 4 different drugs and 5 concentrations after 24 h of exposure.

Mortality								
Drugs	Control	5 μg·mL^−1^	10 μg·mL^−1^	15 μg·mL^−1^	20 μg·mL^−1^	25 μg·mL^−1^	LC_50_	Confidence Interval
Donepezil	0	1 ^ns^	3 ^ns^	4 *	5 *	8 *	>25	20–45
Tacrine	0	2 ^ns^	2 ^ns^	3 ^ns^	6 *	10 *	≤21.2	17.5–31.7
Rivastigmine	0	1 ^ns^	3 ^ns^	5 *	7 *	12 *	≤17.4	15–21
Galantamine	0	4 *	6 *	12 *	15 *	15 *	≤8.3	5.5–12.5

* Means differ by Dunnett’s test (*p* *=* 0.05); ns: non-significant; *n* = 3–15 planarians per group.

**Figure 1:**
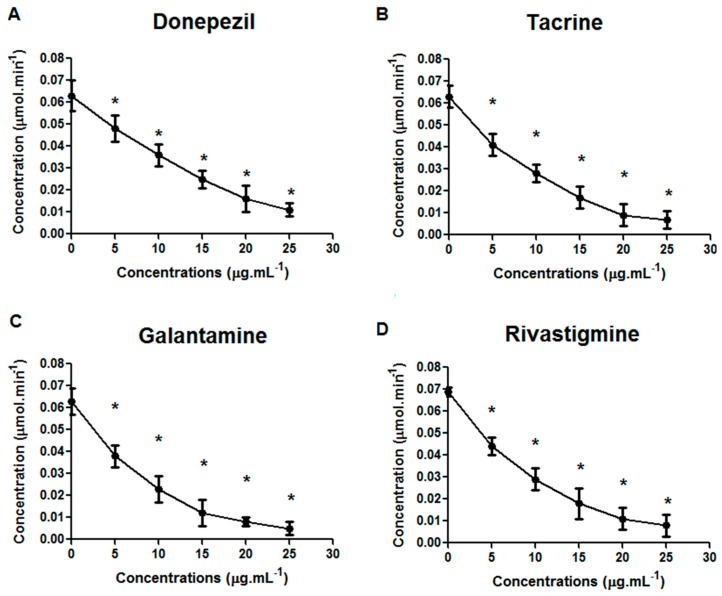
Effects of donepezil (**A**), tacrine (**B**); rivastigmine (**C**) and galantamine (**D**) on the inhibition of acetylcholinesterase in *Dugesia tigrina*, after 24 h of exposure. * Means differ by Dunnett’s test (*p* *=* 0.05). *n* = 3–15 planarians per group.

All drugs inhibited the activity of AChE in *D. tigrina*, in a similar way to trials conducted in experimental models with vertebrates. The lowest concentration did not inhibit the production of AChE compared to other concentrations, but *D. tigrina* was less sensitive to donepezil (0.011 μmol·min^−1^ at concentration of 25 μg·mL^−1^) (Figure 1A). Tacrine and rivastigmine had similar inhibitory activity (0.007 and 0.008 μmol·min^−1^ at a concentration of 25 μg·mL^−1^) (Figure 1B,C), and galantamine provoked more intense inhibitory effects with the production of AchE equal to 0.005 μmol·min^−1^ at a concentration of 25 μg·mL^−1^ (Figure 1D). The activity of AChE is significantly reduced by a concentration above 10 μg·mL^−1^.

The observed effects evidenced the cholinergic action of the drugs on *D. tigrina*, indicating that this species can be utilized for the screening of substances with anticholinesterasic activity. Our results are similar to those found by various authors in studies on the inhibition of AChE activity of the drugs donepezil, tacrine, galantamine and rivastigmine [18,19,20,21,22,23].

The presence of receptors of acetylcholine in Platyhelminthes had already been demonstrated in *D. tigrina*, indicating considerable levels of AChE *in vivo* [24]. Hence, our results showed that *D. tigrina* is emerging as a promising experimental model for the investigation of biochemical and functional interactions among receptor systems of different neurotransmitters and for the pharmacological action of several drugs.

These results are similar to those obtained in mice after administration of anticholinesterase drugs. When administered to rats, they promote changes of cholinesterase activities in the rat blood and tissues, LC_50_ being observed at a concentration of 40.0 mg·kg^−1^ for tacrine [25], 3.0 mg·kg^−1^ for galantamine [26], 25.0 mg·kg^−1^ for donepezil [26] and 75.0 mg·kg^−1^ for rivastigmine [27] after 24 h of administration. These drugs are considered potent acetylcholinesterase inhibitors, have been shown to be effective in biochemical dysfunction induced in rats and are considered pseudo irreversible inhibitors. Although the reversible effect was not the focus of this work, the aforementioned results demonstrate a rapid absorption of the drug by planarians, resulting from the nervous system, which leads to a complete absorption. The exhibition presented by the doses demonstrated inhibitory activity *in vivo* similar to tests conducted with rats.

**Figure 2:**
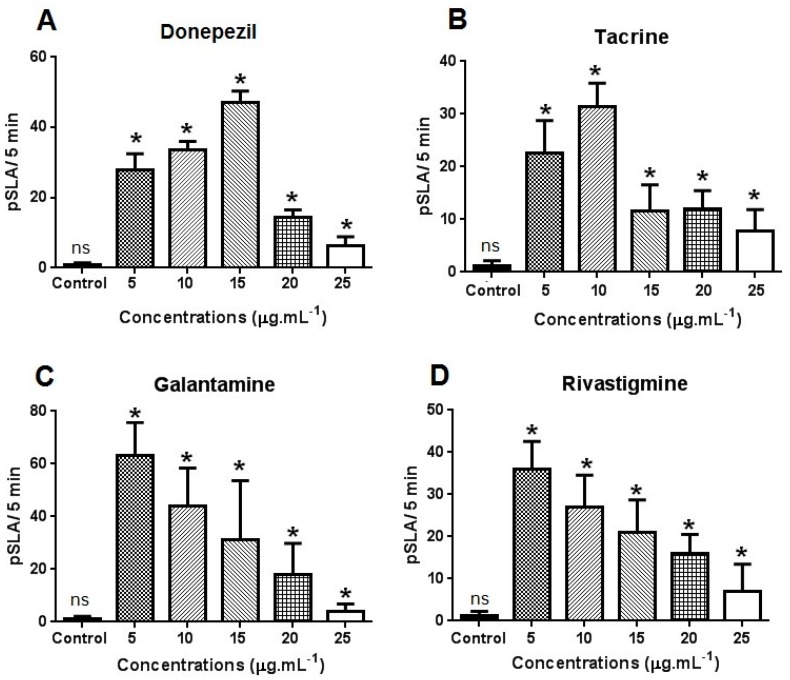
Effects of donepezil (**A**); tacrine (**B**); rivastigmine (**C**) and galantamine (**D**) on planarian seizure-like activity (pSLA) in *Dugesia tigrina*, after 5 min of exposure. Samples were tested in triplicate with 30 planarians in each Petri dish after 24 h of exposure. * Means differ by Dunnett’s test (*p* *=* 0.05).

Figure 2 shows the cumulative planarian seizure-like activity (pSLA) exhibited by *D. tigrina* after exposure to the drugs at different concentrations. A concentration-dependent increase in pSLA was observed in the worms exposed to the different drugs. At a concentration of 25 μg·mL^−1^, the convulsive effects were less pronounced, and *D. tigrina* presented minimal movement after a 5-min exposure under all tested drugs (Figure 2A–D). At the concentration of 25 μg·mL^−1^, the convulsive effects were less pronounced, and *D. tigrina* presented minimal movement after a 5-min exposure under all tested drugs. Locomotor activity was visibly reduced in planarians exposed to the higher concentrations of drugs, and a major effect in locomotor retardation was observed for galantamine and donepezil. As the concentration was increased, the planarians started to exhibit hypokinesia, resulting in a still position. Planarians exposed to 5.0 μg·mL^−1^ of galantamine and rivastigmine exhibited a higher number of pSLA (Figure 2C,D). Donepezil and tacrine had similar convulsive effects, with 48 and 36 pSLA, respectively (Figure 2C,D). PSLA was most affected by galantamine, with 63 convulsive movements under the lowest concentration (5 μg·mL) and four movements at the concentration of 25 μg·mL^−1^ (Figure 2C).

We also observed effects provoked by the drugs, such as convulsions and locomotor retardment, confirming the results described for other pharmacological models [26,28,29,30,31]. Myhrer *et al.* [26] reported that rats treated via intraperitoneal injection with galantamine exhibited a cognitive deficit in terms of reduced preference, also observed for donepezil; the locomotor activity was radically depressed in all groups treated with anticholinesterasics, as well as in combination with procyclidine, but was more prominent in rats injected only with galantamine.

**Figure 3:**
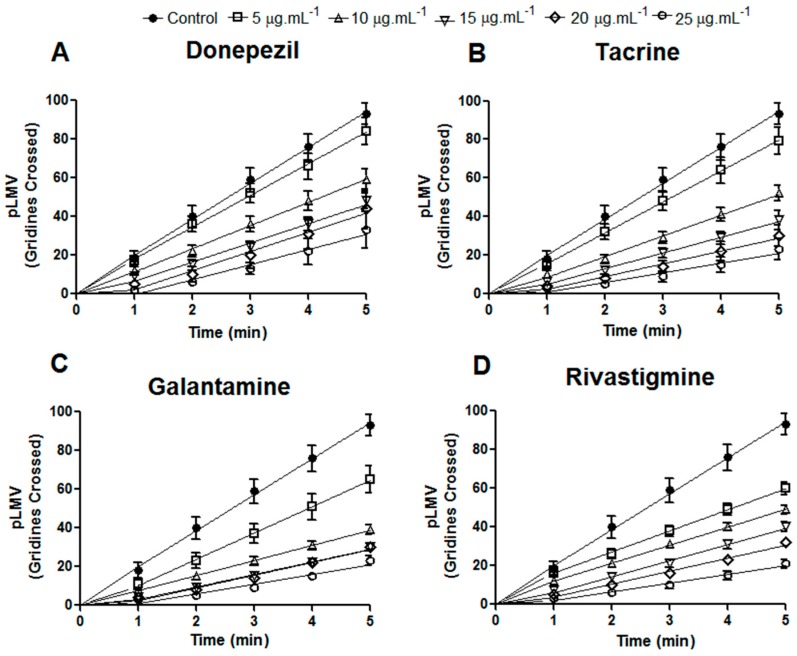
Effects of donepezil (**A**) tacrine (**B**); rivastigmine (**C**) and galantamine (**D**) on motility (planarian locomotor velocity (pLMV)) in *Dugesia tigrina*, after 5 min of exposure. Samples were tested in triplicate with 30 planarians in each Petri dish.

Regarding the number of crossed lines (planarian locomotor velocity (pLMV)), we observed that the drugs reduced the locomotor capacity in *D. tigrina*. Donepezil and rivastigmine presented a lower effect compared to tacrine and galantamine during the observation period of 5 min (Figure 3A,D). Galantamine caused higher retardment of movements in *D. tigrina*, with 10 crossed lines during the 5-min observation (Figure 3C). *D. tigrina* exhibited the characteristic of constant pLMV over the observation period, with 18 grid lines per minute in the control with artificial lake water. The concentration of 5 μg·mL^−1^ did not cause a significant reduction by the tested drugs, with a mean of 8–17 lines crossed per minute (Figure 3A–D). A low convulsant activity under the highest doses was similar to the loss of motility at the same concentrations. Galantamine most affected *D. tigrina* in pSLA and pLMV (Figure 2 and Figure 3C). The results of the locomotion delay can be explained by a non-uniform distribution of the drugs used, as demonstrated for tacrine, talantamine and physostigmine; these accumulate in the brain tissue of small animals [32], and this accumulation (or bioavailability) could be a reason for the different concentrations for inhibition *in vivo*.

The inhibitors of AChE can provoke convulsions induced by nervous agents and may have lethal effects. Myhrer *et al.* [26] reported that physostigmine (0.1 mg·kg^−1^), galantamine (3 mg·kg^−1^), huperzine (0.5 mg·kg^−1^) and donepezil (2.5 mg·kg^−1^) provoke locomotion retardment in all treated groups, with more prominent effects for galantamine, as we also observed in our study.

It is probable that the increase in cholinergic activity in the brain is related to an initial phase of convulsions [33,34], while the established convulsions are probably associated with increased glutamatergic activity, leading to neural damage predominantly in the hippocampus, pyriform cortex and entorhinal cortex [34,35].

In future studies, it will be important to clarify the possible role of the activity of subclinical apprehension in the neuropathological syndrome induced by the treatment with the tested drugs. We considered it probable that the animals, including those that remain relatively motionless with behavioral signs of minimal convulsive activity, treated with different concentrations of drugs, in particular galantamine, underwent discharges of the convulsive crisis, which increase the synaptic concentrations. Other studies demonstrated that donepezil causes hypothermia, hypoactivity and diarrhea after administration of 1.25 mg·kg^−1^. Such symptoms may be associated with lethality, being implications associated with disturbances of the nervous system [36]. The administration of tacrine to treat Alzheimer’s disease causes hepatotoxicity in 30%–50% of patients, indicated by increased levels of transaminases [37].

Our results add to other studies that demonstrate the use of *D. tigrina* in pharmacological models, such as trials on regeneration and alterations in glycolytic metabolism [38], chemo-convulsants [15], interactions of acetylcholine and dopamine [39], the increase of mobility and the proliferation of stem cells [40], morphogenesis [14], bipolar regeneration [14,17], convulsive activity [15], locomotion retardment [41] physical dependence [42] and the production of serotonin [43].

These results suggest that pLMV can serve as a practical and valuable model to complement studies on the inhibition of AChE in planarians, which could be utilized as an *in vivo* model. The models of rats most utilized only include the inhibition of AChE in rat brains, indicated by various already known drugs [33,34,35,39], beside the reduction in locomotor ability [44,45]. The planarian model has the advantages of an invertebrate over a vertebrate model. Raffa *et al.* [44] have demonstrated that the planarian model gives a quantifiable approach, sensitive for studies on dopamine receptors, and stated that this method can be applied to study pharmacological and toxic agents and drugs of abuse. Planarians are sensitive due to a diffuse nervous system. Therefore, the behavioral sensitization in mammals, including human beings, is affected by the administration type and experimental environment, and some results may not have any relation to drugs that are difficult to eliminate. More simple animals and those less susceptible to such confounding influences can be advantageous substitutes for studies concerning the nervous system. Rawls *et al.* [45] reported that the inability to control those factors associated with sensitivities can hinder the interpretation of results in mammals.

Changes in behavior have been little studied in invertebrates, and planarians became attractive organisms to investigate the process of behavioral sensitization. Even more importantly, planarians exhibit hyperactivity or hypoactivity after acute exposure to drugs, allowing quantifying alterations in behavior.

Planarians have key substrates that were identified as primary mediators of behavioral sensitization [45], they being: (1) establish to studying behavioral sensitization as a simple biological parameter that is sensitive, reproducible, quantifiable, cost-effective and time-effective; (2) to minimize interpretive complications related to pharmacokinetic factors, such as drug absorption, distribution, metabolism and elimination; (3) to precisely identify the drug concentration, as opposed to just the dose, which is less amenable to rigorous quantitative analysis; (4) to continuously expose the animal to a drug (soaking it in a Petri dish), a feat accomplished easily with planarians because of their aquatic nature, thus offering a distinct advantage over mammalian models that rely on multiple injections or mini-pumps to achieve constant drug concentrations; (5) to reduce the impact of higher-order confounding influences, such as handling, familiarity of the testing environment, strain, stress from procedures and anticipation of drug administration; (6) to adapt the model of behavioral sensitization to translational use, such as preclinical testing and *in vivo* screening of very small amounts, allowing one to investigate its potential in CNS; (7) to study the effects of poly-drug exposure, particularly as related to screening compounds for their efficacy against the adverse effects produced by simultaneous exposure; and (8) to reduce the use of mammals in research.

Hence, our study reinforces the potential use of *D. tigrina* as experimental models of inhibition of acetylcholinesterase in the search for new substances that could be utilized in the treatment of Alzheimer’s disease. Models that utilize *D. tigrina* are even more attractive for the easiness of maintaining and manipulating them under laboratory conditions and are of low cost in comparison to other models of test animals.

## 3. Experimental Section

### 3.1. Collection and Acclimation of D. tigrina

Samples of *D. tigrina* were collected from the lake of the Barigui Park, city of Curitiba, PR, Brazil, at the coordinates 25°25′34.52′′ S, 49°18′27.03′′ W. The planarians were taken to the laboratory, and individuals *ca.* 6–10 mm were separated, enclosed in plastic trays with artificial lake water (6 mM NaCl, 0.1 mM NaHCO_3_, 0.6 mM CaCl_2_) and the pH adjusted to 6.9 with a solution of NaOH 1 N [31]. Next, the tray was taken to a BOD-Biological Organisms Development (Model MA-403, Marconi) chamber, under adequate conditions of temperature (20 °C) and relative humidity (80%) [46], for 24 h before the bioassays.

### 3.2. Evaluation of Mortality

The drugs galantamine, donepezil, rivastigmine and tacrine were chosen for being utilized in treatment of Alzheimer’s disease. All drugs were obtained from Sigma Aldrich^®^, and the concentrations of 5, 10, 15, 20 and 25 μg·mL^−1^ were prepared for utilization in the trials, being previously dissolved in DMSO 0.1%, followed by distilled water.

The choice of concentrations was based on a pre-mortality test with concentrations ranging from 5–100 μg·mL^−1^. From the pre-test, we decided to use concentrations of 5–25 μg·mL^−1^. Such concentrations were chosen because >25 μg·mL^−1^ caused 100% mortality. Drugs were diluted in DMSO, because they are not soluble in other solvents. DMSO concentrations higher than 0.1% caused changes in the behavior of planarians and were therefore discarded.

The pH of the solutions was adjusted to 6.9 with KOH 1 N using a pH meter (MPA 210, Tecnopar, Curitiba, PR) and put in plastic pots of 5 mL. Trials were performed in three Petri dishes containing 30 planarians on each plate. In the control, the same conditions of the tests conducted for the drugs were used, only DMSO solution 0.1% being added to the water.

After the acclimation period, 15 individuals were placed in each Petri dish (9 cm diameter) and included in the test solutions with the different concentrations, totaling 1080 (thousands eighty) individuals utilized for the trial, and exposed during 24 h (standard exposure time for acute toxicity), where 3 replicates were used for each concentration. All time points chosen for these tests are based on other reported studies for other aquatic organisms [47,48,49,50,51,52]. After this period of exposure, the number of dead individuals was recorded. In this trial, we considered dead the individuals that did not respond to stimuli of light or when touched. The collected data were submitted to probit analysis, to calculate the results regarding LC_50_, which was determined by nonlinear regression using the Probitos program [53].

### 3.3. Preparation of the Enzymatic Extract

To evaluate the enzymatic activity, the planarians were submitted to the test solutions during a period of 6 h; then, drugs were removed, and the planarians were gently washed with artificial lake water, at least three times. Next, the planarians were placed in a porcelain mortar and homogenized in 900 μL of 0.1 M buffer phosphate (pH 7.5) containing 0.1% (*v*/*v*) of Triton X-100 and 100 μL of protease inhibitors (Sigma Aldrich^®^, St. Louis, MO, USA) and centrifuged at 12,000 rpm, 20 min at 4 °C [54]. Supernatants were gathered and utilized as source of enzymes for the determination of AChE and total proteins. The concentration of total proteins of each enzymatic extract was determined, using bovine serum albumin (Sigma Aldrich^®^) as a standard [55].

### 3.4. Determination of Acetylcholinesterase

The activities of acetylcholinesterase (AChE) were performed according to the modified procedure of Ellman [56]. The activity of AChE was determined after the reaction of the Ellman reagent (DTNB-5,5′-Dithiobis-(2-Nitrobenzoic Acid)) and the thiocholine generated by enzymatic hydrolysis of acetylthiocholine iodide (ATCH), respectively, by the addition of 100 μL of enzymatic extract (1.3–1.8 mg of protein of enzyme extract), 800 μL of buffer PBS 1 × (pH 7.4), 100 μL of DTNB (3,3′-Dithio-bis (6-nitrobenzoic acid) and 200 μL of ATCH 1 mM. The reagents were obtained from Sigma Aldrich^®^. Next, 300 μL of the samples were transferred to 96-well microplates and the absorbance read at 412 nm (thermo scientific multiscan FC model microplate reader). The results were expressed in μmol·min^−1^, to which all described enzymes the enzymatic extracts were added, with the specific reagent solutions for each enzymatic dosage.

### 3.5. Measures of Seizure-Like Activity

The convulsant activity by means of measurements of seizure-like activities (planarian seizure-like activity (pSLA)) is defined as asynchronous paroxysms resulting in a sudden interruption of the spontaneous normal locomotor activity, and observed convulsive movements, such as posture similar to C-like, screw-like, snake-like and walnut position, hyperkinesia and hypokinesia, which are very different from normal locomotor movements [41].

The duration of each behavior is approximately 1 s. To measure pSLA, the individuals were placed in glass Petri dishes (60 × 15 mm) containing the solutions at different concentrations of the drugs and artificial lake water. The exposure time was 5 min. The accumulation of pSLA 5 min was calculated as the number of observed behavior per minute, during the 5-min exposure, according to the parameters established by Raffa *et al.* [44]. Each planarian was tested only once to determine the convulsive effect [15], and the counting of pSLA was done double-blind.

### 3.6. Motility Measurement (pLMV)

The locomotion delay in *D. tigrina* was evaluated by means of trials of pLMV (planarian locomotor velocity). In this procedure, 15 individuals were individually placed in Petri dishes (9 cm diam) containing 5.0 mL of solution of the drugs in the same concentrations utilized in the previous trials. The pLMV and pSLA provided a stable baseline behavior against which to compare the action of pharmacologic test agents and, after 5 min of exposure, did not show changes in behavior [44].

The exposure time to the drug followed the protocol of Raffa *et al.* [44]. After 5 min of exposure, the planarians were washed 3 times with distilled water and placed in Petri dishes with 5.0 mL of chlorine-free water. The Petri dishes were placed on millimeter-ruled paper with a 0.5-mm distance, and the pLMV was quantified by the observation of the accumulated number of lines crossed by each planarian per minute. Each planarian was used only once [44].

### 3.7. Statistical Analysis

In the mortality assay, the experimental design was a completely randomized factorial 4 × 5, with an additional treatment (control), totaling 21 treatments (4 × 5 + 1), with three replicates for each treatment. All values were expressed as the mean ± SEM, and for the comparison of the results regarding the number of dead individuals, the AChE levels were assessed utilizing analysis of variance (ANOVA) followed by a *post hoc* Dunnett’s test for the analysis of the detailed data. The level of significance was *p* *=* 0.05. The locomotor velocity (pSLA and pLMV) is expressed as the mean (±SEM) of the cumulative number of grid lines crossed by each planarian per minute. Relative potency was calculated from linear regression analysis of individual dose-response curves, according to the protocol established by Raffa *et al.* [44].

## 4. Conclusions

These results demonstrate that *D. tigrina* can be used as a model for the investigation of AChE inhibitory substances, because they are low maintenance with respect to manipulating them under laboratory conditions and are low cost in comparison to other models of test animals.
